# Vitamin D supplementation and its influence on muscle strength and mobility in community‐dwelling older persons: a systematic review and meta‐analysis

**DOI:** 10.1111/jhn.12394

**Published:** 2016-07-27

**Authors:** H. Rosendahl‐Riise, U. Spielau, A. H. Ranhoff, O. A. Gudbrandsen, J. Dierkes

**Affiliations:** ^1^ Department of Clinical ScienceUniversity of BergenBergenNorway; ^2^ Institute of Agricultural and Nutritional SciencesMartin‐Luther University Halle‐WittenbergHalleGermany; ^3^ Department of Clinical MedicineUniversity of BergenBergenNorway; ^4^ Kavli Research Center for Geriatrics and DementiaHaraldsplass Deacon HospitalBergenNorway

**Keywords:** ageing, gait, hand strength, muscle strength, vitamin D

## Abstract

**Background:**

It has been suggested that vitamin D status or supplementation is important for maintaining or improving muscle strength and mobility in older adults. The study results, however, do not provide consistent results. We therefore aimed to summarise the available evidence systematically, including only studies conducted in community‐dwelling older persons.

**Methods:**

A systematic search of the literature was performed in April of 2016. The systematic review includes studies that used vitamin D with or without calcium supplementation as the exposure variable and various measurements of muscle strength and mobility. The meta‐analysis was limited to studies using hand grip strength (HGS) and timed‐up‐and‐go test as the outcome variables.

**Results:**

A total of 15 studies out of 2408 articles from the literature search were included in the systematic review, providing 2866 participants above the age of 65 years. In the majority of studies, no improvement in muscle strength and mobility was observed after administration of vitamin D with or without calcium supplements. In the meta‐analysis, we observed a nonsignificant change in HGS [+0.2 kg (95% confidence interval = −0.25 to 0.7 kg; seven studies)] and a small, significant increase in the timed‐up‐and‐go test [0.3 s (95% confidence interval = 0.1 to 0.5 s; five studies)] after vitamin D supplementation. The meta‐analyses showed a high degree of heterogeneity between the studies.

**Conclusions:**

In conclusion, we observed no improvement in muscle strength after the administration of vitamin D with or without calcium supplements. We did find a small but significant deterioration of mobility. However, this is based on a limited number of studies and participants.

## Introduction

Among other changes in body composition and functions, ageing involves a decrease in skeletal muscle mass and strength, and consequently also in mobility 1. This loss of muscle mass and strength is called sarcopenia and is associated with falls, fractures, immobilisation and mortality. Sarcopenia has an estimated prevalence of 5–13% in 60–70 year olds and 11–50% in persons older than 80 years 2. These numbers demonstrate that declining muscle mass and strength are significant and age‐dependent problems in older persons. Early and continuous interventions may be key to limiting this decline and preserving both muscle mass and strength. A number of dietary measures (supplementation with protein, energy, or *n*‐3 polyunsaturated fatty acids, micronutrient supplementations) 3, 4, 5, 6, 7, 8 and exercise interventions 9, 10 or their combinations have been tested 11, 12, 13, 14. Among these interventions, supplementation with vitamin D has been promoted as having positive effects in older persons with respect to the risk of falls and fractures 15, 16. Usually, meta‐analyses investigating the effect of vitamin D on the risk of falling include studies using vitamin D either with or without calcium supplements. Therefore, it is impossible to conclude whether vitamin D supplementation would be effective on its own or not. This can be regarded as a serious limitation of previous randomised controlled trials (RCTs) and meta‐analyses and can also lead to inconsistent conclusions 17. In a newer meta‐analysis investigating primarily the effect of vitamin D on hip fractures in older adults 18, however, it was concluded that vitamin D with calcium was effective in preventing fractures, although the effect of vitamin D without calcium was not significant. An increased risk of falls can be seen as a consequence of low muscle strength and mass 19. It has been estimated that the risk of falls increases with age and the presence of frailty and falls are a common cause of fractures in old adults 20. The incidence of falls is difficult to measure, and falls may also have many other causes. Direct measurements of muscle strength and mobility are therefore required to study the effect of vitamin D.

Vitamin D deficiency is widespread in adult and older populations 21, even in populations without other overt nutrient deficiencies 22. Vitamin D supplementation is usually combined with calcium supplementation, aiming to ensure sufficient calcium from the diet during vitamin D supplementation 23, 24. Vitamin D may exert its influence on skeletal muscle cells by the presence of the vitamin D receptor, and may also be needed for optimal muscle function 21 and adequate protein synthesis. In observational studies, an adequate 25(OH)D concentration was associated with better musculoskeletal function and muscle strength 25, 26. However, the optimal level of 25(OH)D in older persons is still unknown, although it is suggested to be ≥65 nmol L^−1^ 27.

Older people represent a very heterogeneous group. In general, community‐dwelling older persons are younger and in better health and a better functional state compared to institutionalised individuals, although a high prevalence of comorbidities of chronic diseases may be present. In particular, institutionalised older persons show higher degrees of frailty, are more dependent in activities of daily living, and have a higher prevalence of cognitive decline. It is therefore justified to distinguish between these two groups when analysing dietary measures aimed at reducing the decline in muscle strength and mobility, although a number of studies did not make this distinction 28, 29, 30, 31, 32, 33. However, vitamin D supplementation for the prevention of loss of muscle strength and mobility has not been established in either group. In addition, studies vary in their design, the type and length of the intervention, and the outcomes because different measurements of muscle strength and mobility have been used, and there is no common protocol for these assessments.

The objective of this systematic review and meta‐analysis was to investigate the effects of vitamin D supplementation (with or without calcium) in community‐dwelling older subjects on muscle strength and mobility, based on the results from RCTs.

## Materials and methods

### Data sources and search strategy

Relevant studies were identified by a systematic search of current literature using PubMed, Embase, Medline, Web of Science and the Cochrane Library, followed by a manual search of the extracted articles and existing reviews. The clinical trial registry ‘ClinicalTrials.gov’ was searched for unpublished trials. The search covered the period up to 13 April 2016. The search terms are presented in the Supporting information (Appendix S1).

**Figure 1:**
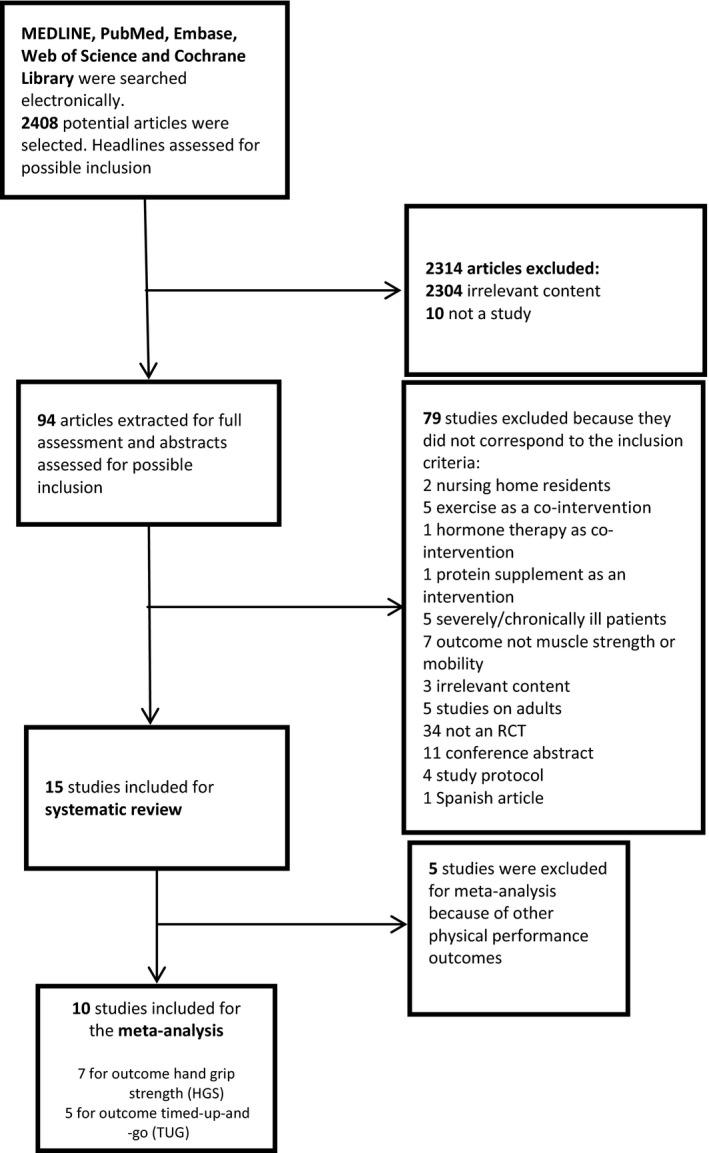
Flow chart of the selection of studies on the effect of vitamin D supplementation with or without calcium supplements on muscle strength and mobility in the present systematic review and meta‐analysis. RCT, randomised controlled trial.

The inclusion criteria are stated in Table 1. Differences in dosage, frequency, mode of delivery or the form of the vitamin D supplementation were not a cause for exclusion. It became apparent that only the outcomes comprising hand grip strength (HGS) and timed‐up‐and‐go (TUG) were investigated in a sufficient number of studies to perform a quantitative meta‐analysis, whereas other outcomes of muscle strength and mobility were only included in the systematic review. Two of the authors (HRR, JD) screened the article titles and abstracts to identify studies that were suitable for inclusion. Ninety‐four articles were read as full papers, and 15 studies were selected for systematic review (Fig. 1). These 15 studies also included one identified by searching clincialtrials.gov 34. The 15 articles were evaluated by all authors. Three other studies identified from clinicaltrials.gov were either still ongoing or a study protocol, and were therefore not included.

**Table 1:** Overview of the inclusion criteria for the present systematic review and meta‐analysis

Design	Randomised controlled trials
Participants	Older persons >65 years of age Humans Community‐dwelling
Intervention	Vitamin D supplementation – all forms and all doses, with or without calcium supplements or dietary advice
Comparator	Low dose of vitamin D or vitamin D metabolites or placebo, with or without calcium supplement
Outcome measures	
Systematic review	Measures of muscle strength and mobility
Meta‐analysis	Hand grip strength (HGS)
	Timed‐up‐and‐go test (TUG)

The various outcomes used in these studies are described in the Supporting information (Appendix S2). The overall quality of the full articles was assessed using the CONSORT statement checklist for assessing quality of randomised clinical trials 35. The CONSORT statements are summarised in the Supporting information (Appendix S3).

### Data collection

All relevant information was extracted from eligible studies and is available in Table 2. Any other information necessary for the review, such as potential covariates to the RCT (e.g. the season in which the RCT took place and any ultraviolet‐B exposure), the dropout rate and compliance, was also noted when reported.

**Table 2:** An overview of the studies included in the systematic review and meta‐analysis

Study	Sample size (sex), *n*	Age (years)	Serum 25(OH)D status at baseline (nmol L^−1^) [mean (SD)]	Method used for analysing 25(OH)D	Study duration	Study design	Comparator	Form and dosage of vitamin D	Calcium supplement (mg)	Physical performance measure
Bischoff‐Ferrari (2012) 42	20 (females)	C: 63.45 (7.78) I: 59.48 (6.27)	C: 35.45 (9.03)^c^ I: 30.7 (10.2)^c^	HPLC‐MS/MS	4 months	Randomised, double‐blinded trial	C: 800 IU D_3_ day^−1^ C: 5600 IU D_3_ week^−1^	I: 20 μg HyD day^−1^ I: 140 μg HyD week^−1^	Non	TUG (3 m) Knee extension Knee flexion strength Repeated sit‐to‐stand
Ceglia (2013) 34	21 (females)	C: 80 (5) I: 76 (4)	C: 48.3 (8.8) I: 43.6 (10.3)	RIA (DiaSorin Inc., Stillwater, MN, USA)	4 months	Randomised, double‐blind, placebo‐controlled Single centre	Placebo	4000 IU (D_3_) day^−1^, oral	Non (dietary intake was assessed)	Knee extension SPPB (incl. 4 m TUG)
Dhesi (2004) 48	139	C: 76.6 (6.1) I: 77.0 (6.3)	C: 25.0^b^ (23.8–26.3) I: 26.8^b^(25.5–28.0)	IDS Gamma‐B 25‐OH immunoassay (IDS, Tyne & Wear, UK)	6 months	Randomised, double‐blind, placebo‐controlled	Placebo	600 000 IU (D_2_) × 1 bolus inj.	Non	Quadriceps strength AFPT
Glendenning 2012 49, a	686 (females)	C: 76.5 (4) I: 76.9 (4)	C: 66.5 (27.1) I: 65.0 (17.8)	Liaison method (DiaSorin Inc.)	3, 6 and 9 months	Randomised, double‐blind, placebo‐controlled	Placebo	150 000 IU (D_3_) every 3 months, oral	Advice: 1300 (supp./diet)	Grip strength (kg) TUG (3 m)
Grady (1991) 51, a	98	C: 78.9 (5.4) I: 79.4 (5.4)	C: 65.7 (51.4) I: 60.4 (35.3)	Microassay by Reinhart *et al*., 1984	1, 2, 4, 8, 12, 18 and 24 weeks	Randomised, double‐blind, placebo‐controlled	Placebo	0.5 μg (1,25‐dihydroxyvitamin D_3_) day^−1^, oral	Non (dietary intake was assessed)	Grip strength (kg) Leg muscle strength
Janssen (2010) 53, a	70 (females)	C: 79.2 (6.7) I: 82.4 (4.9)	C: 34.3 (11.5) I: 32.6 (11.6)	NA	6 months	Randomised, double‐blind, placebo‐controlled	Placebo	400 IU (D_3_) day^−1^, oral	500	Knee extension Hand grip strength (kg) LEP TUG (4 m) Modified Cooper test
Kenny (2003) 52, a	65 (men)	76 (4)	C: 60 (18)^c^ I: 65 (18)^c^	Competitive protein binding (Endocrine Science Inc., Calabasas Hills, CA, USA)	6 months	Randomised, double‐blind, placebo‐controlled	Placebo	1000 IU (D_3_) day^−1^, oral	500	Leg extension strength Grip strength (kg) SPPB (incl. 3 m TUG)
Lagari (2013) 44, a	86	73.4 (6.4)	82.5 (25.0)^c^	LC/MS/MS	6 months	Randomised, double‐blinded trial	400 IU D_3_ day^−1^, oral	2000 IU (D_3_) day^−1^, oral	Calcium supplements was assessed	Grip strength (kg) Gait speed
Lips (2010) 43	593	C: 77.6 (6.6) I: 78.5 (6.2)	C: 35.3 (13.8)^c^ I: 34.3 (11.0)^c^	Reversed phase HPLC by Lensmeyer *et al*., 2006	16 weeks	Randomised, double‐blind, placebo‐controlled Multicentre	Placebo	8400 IU (D_3_) week^−1^, oral	500 for those with dietary intake <1000 mg	SPPB
Pfeifer (2009) 45, a	242	77 (4)	C: 54 (18) I: 55 (18)	RIA (Immunodiagnostic Systems, Boldon, UK)	12 and 20 months	Randomised, double‐blind, placebo‐controlled Multicentre	Placebo	800 IU (D_3_) day^−1^, oral	1000	Quadriceps strength (isometric leg extensor strength) TUG (3 m)
Pirotta (2015) 50, a	26	C: 71.5 (5.7) I: 66.1 (4.0)	C: 48.5 (11.1) I: 46.4 (11.4)	Liaison method (DiaSorin)	10 weeks	Randomised, double‐blind, placebo‐controlled	Placebo	2000 IU (D_3_) day^−1^, oral	Non	Knee extensor Stair climbing power FSST TUG (3 m)
Songpatanasilp (2009) 46	72 (females)	70.60 (4.30)	69.98 (19.18)^c^	RIA (DiaSorin)	12 weeks	Randomised placebo‐controlled trial	Placebo	0.5 mg (20 000 IU) (alfacalcidiol) day^−1^, oral	1500	Quadriceps strength (isokinetic dynamometer)
Wood (2014) 38, a	305 (females)	63.8 (2.2)	Normal: 34.3 (14.7) Overweight: 33.9 (14.3) Obese: 32.4 (16.3)	LC/MS/MS (Chromsystems, UK)	12 months (bimonthly study visits)	Randomised, double‐blind, placebo‐controlled	Placebo	I: 400 IU (D_3_) day^−1^, oral I: 1000 IU (D_3_) day^−1^, oral	Non	Grip strength (kg)
Xia (2009) 55, a	142 (females)	C: 70.4 (3.6) I: 70.4 (3.9)	NA	NA	6 and 12 months	Randomised, multicentre, open‐label, placebo‐controlled	125 IU (Calcitriol) day^−1^, oral	125 IU + 0.25 μg (Calcitriol) day^−1^, oral	600/600	Grip strength (kg) FTFFT
Zhu (2010) 47, a	302 (females)	C: 77.0 (4.8) I: 77.6 (4.2)	C: 44.3 (13.0)^c^ I: 45.3 (12.5)^c^	RIA (DiaSorin)	6 and 12 months	Randomised, double‐blind, placebo‐controlled	Placebo	1000 IU (D_2_) day^−1^, oral	1000	TUG (3 m) Lower limb muscle strength (ankle, knee, hip)

a Included in the meta‐analysis.

b Geometric mean and 95% confidence interval.

c Calculated to nmol L^−1^using coefficient of 2.5.

AFPT, aggregate functional performance time; C, control; FSST, the four square step test; FTFFT, Five‐times‐sit‐to‐stand‐test; HPLC, high‐performance liquid chromatography; HyD, 25‐hydroxyvitamin D3; I, intervention; LC/MS/MS, liquid chromatography, tandem mass spectrometry; LEP, Leg extension power; NA, not available; RIA, radioimmunoassay; SPPB, Short Physical Performance Battery; TUG, timed‐up‐and‐go.

### Statistical analysis

**Figure 2:**
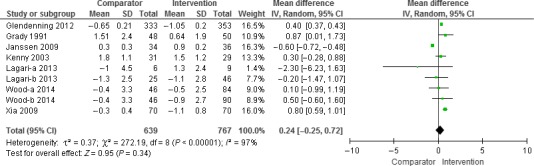
Results of the meta‐analysis of the effect of vitamin D supplementation with or without calcium on hand grip strength (kg) (*n* = 7 studies). The results were obtained using a random effects model. One study reported results for men and women separately (Lagari a – men and Lagari b – women). For one study, we have divided the comparator group in two (Wood‐a and Wood‐b). CI, confidence interval.

**Figure 3:**
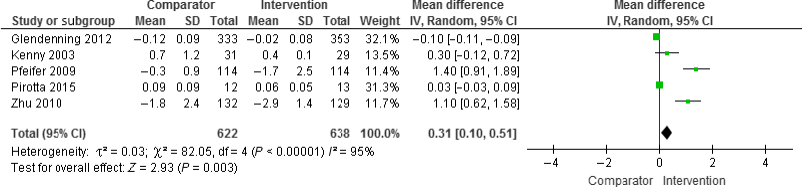
Results of the meta‐analysis of the effect of vitamin D supplementation with or without calcium on timed‐up‐and‐go (TUG) (*n* = 5 studies). The results were obtained using a random effects model. CI, confidence interval.

Other outcomes than TUG and HGS were reviewed narratively as a result of the low number of studies evaluating these outcomes. Only for TUG and HGS did we find more than three studies for a quantitative meta‐analysis. We used RevMan, version 5.3 (Cochrane collaboration) 36 for the analysis, with the outcome being represented by Forrest plots (Figs 2 and 3). Weighted mean differences for vitamin D versus placebo/control were calculated by subtracting the mean of the outcome of interest at the end of the study from the mean at baseline. Standard deviations (SDs) of the differences were calculated using a formula given in the *Cochrane Handbook* 37, applying correlation coefficients of 1.0 for the HGS and 0.8 for the TUG‐test. Because significant heterogeneity was observed between studies with a fixed effect model, we finally applied a random effects model. Studies that included more than one intervention group 38 were treated by dividing the number of subjects in the control group by the number of comparisons at the same time as retaining the mean (SD) of the change according to the *Cochrane Handbook* 37.

Subgroup analysis was conducted with predefined study characteristics: baseline vitamin D status, oral administration of the supplement, daily dose of vitamin D, placebo group, supplementation with vitamin D_2_ or D_3_, and advice on calcium supplementation to explore possible reasons for the observed heterogeneity 39, 40.

## Results

### Search results

As per the Quality of Reporting of Meta‐analyses (QUOROM) 41 flow diagram (Fig. 1), 15 out of 2408 studies were included in the systematic review and 10 of the 15 were eligible for the meta‐analysis.

### Narrative review

#### Study characteristics

We included a total of 15 studies, with a total of 2866 participants aged 65 years and older. Two studies were included with an average age of the participants of 63.8 years (range 60–70 years) 38 and 61.5 years (range 50–70 years) 42, whereas the average age in the other studies included was between 70 and 80 years. The ratio of men to women was approximately 1 : 9 (229/2044), not including one study that did not specify the participants’ sex (*n* = 593) 43. The studies were conducted in Australia, China, Thailand, USA, Canada and Europe (Germany, Austria, Netherlands, Switzerland, Scotland and the UK). One study was a multicentre study, with centres in North America, Mexico and Europe 43. The participants were all community‐dwelling older persons, who were generally in age‐related good health, and a history of chronic conditions such as cardiovascular disease was usually not treated as an exclusion criterion. All studies excluded patients with acute diseases. In general, underlying diseases serving as exclusion criteria were not sufficiently described.

The vitamin D status was measured as the 25(OH)D concentration in 13 of the 15 studies, with chromatographic methods being used in four of them 38, 42, 43, 44. Other studies used radioimmunoassay (DiaSorin Inc., Stillwater, MN, USA) 34, 45, 46, 47, IDS Gamma‐B 25‐OH immunoassay (IDS, Tyne & Wear, UK) 48, Liaison method (DiaSorin Inc.) 49, 50 microassay as described per Reinhardt *et al*., 1984 51, competitive protein binding (Endocrine Science Inc., Calabasa Hills, CA, USA) 52. One study did not report what method had been used 53.The mean baseline serum 25(OH)D concentration ranged between 25 and 82 nmol L^−1^ 54. The average concentration exceeded the cut‐off level of 50 nmol L^−1^ for defining a sufficient status, in six of the 15 studies 44, 45, 46, 49, 51, 52 and, on average, was below that value in eight of them 34, 38, 42, 43, 47, 48, 50, 53 and was not reported in one study 55.

All of the studies selected declared that they had a randomised parallel design. Three did not have a placebo group but used a low dose of vitamin D_3_ (400 IU day^−1^) as a control 44, 55 or the recommended dose for elderly (800 IU day^−1^) 42. One study did not state whether it had been blinded or not 45. One was a randomised, multicentre, open‐label, placebo‐controlled study 55. The randomisation process was usually not sufficiently well described.

Seven of the 15 studies included calcium with vitamin D supplement or placebo 43, 45, 46, 47, 52, 53, 55. Three studies assessed the intake of supplements or dietary calcium intake 34, 44, 51. One study specified calcium supplementation or dietary intake of calcium of 1300 mg 47. Two studies assessed the overall nutrient intake 38, 50. One study excluded participants using high‐dose (>600 mg) calcium supplements 42. One study did not consider calcium intake at all 48.

Both season and latitude can be important covariates as a result of internal vitamin D production by ultraviolet‐B radiation 21. The season of blood withdrawal was not stated in six of the 15 studies 34, 42, 50, 53, 55 and the geographic latitude was stated in three 38, 45, 47. Other covariates of vitamin D status (body mass index, ethnicity, smoking) were usually not considered specifically. The exception was one study that specifically investigated the effect modification of different body mass index groups (normal, overweight and obese) in participants with Caucasian ethnicity 38.

The dropout rate was given in 13 of the studies, and ranged from 0% to 22%. In one of these studies, the dropout rate was given in another publication 56. In one study, the authors noted that the dropout rate was low, without further details 34; in one study, the dropout rate was not reported at all 44.

Compliance was not mentioned in four of the 15 studies 43, 46, 52, 55. In eight of the studies, the compliance was reported to be better than 80% 38, 42, 44, 47, 49, 50, 51, 53. One study used bolus injections, so that 100% compliance may safely be assumed 48. In one study, the compliance rate was 100% for the participants completing the study 49. In one study, the authors stated that a daily compliance calendar had been kept but did not report on compliance 34.

Different metabolites of vitamin D were used, including vitamin D_2_ 47, 48, vitamin D_3_ 34, 38, 43, 44, 45, 49, 50, 52, 53 1,25‐ dihydroxyvitamin D_3_ 51, 55, alfacalcidiol 46 or 25(OH)D_3_ 42 with various doses, administration routes and treatment periods, ranging from bolus injection of 600 000 IU of vitamin D_2_ 48; 1000 IU daily oral dose of vitamin D_2_ 47; oral vitamin D_3_ in doses of 150 000 IU every 3 months 49; weekly oral dose of 8400 IU vitamin D_3_ 43; and daily oral supplement in doses ranging from 400 to 4000 IU vitamin D_3_ 34, 38, 44, 45, 50, 52, 53. The studies that used 1,25‐dihydroxyvitamin D used a daily oral dose of 1,25‐dihydroxyvitamin D (0.5 μg) compared to placebo 51 or 125 IU (vitamin D_3_) compared to 0.25 μg calcitriol 125 IU^−1^ 55. One study included four groups comparing different doses of vitamin D3 (800 IU day^−1^ and 5600 IU week^−1^) with 25(OH)D_3_ (20 μg day^−1^ and 140 μg week^−1^) 42.

An overview of the methodological quality of the studies is presented in the Supporting information (Appendix S3).

### Study outcomes

Measurements of physical performance outcomes are not standardised, and various methods had been used in the clinical studies (see the Supporting information, Appendix S2). Four studies used complex outcome measurements such as the Short Physical Performance Battery (including TUG) 34, 43, 52 and the aggregate functional performance time 48.

Studies that used single outcome measurements included the knee extension test 34, 42, 50, 53. Three studies used quadriceps strength (using various protocols thus precluding a formal meta‐analysis) 45, 46, 48, HGS 38, 44, 49, 51, 52, 53, 55 and the 3‐m TUG 42, 45, 47, 49, 50, 52. Other available physical outcome measures were the 4‐m TUG, leg muscle strength, leg extension strength, gait speed, Five‐Times‐Sit‐to‐Stand‐Test, leg extension power, modified Cooper test, stair climbing power, the four square step test, repeated sit‐to‐stand, knee flexion strength and lower limb muscle strength.

The authors of nine studies concluded that supplementation with vitamin D and/or calcium did not have any beneficial effect on mobility and/or muscle strength 34, 38, 43, 44, 48, 49, 51, 52, 53. In six studies, they found an improvement in mobility and/or muscle strength 42, 45, 46, 47, 50, 55. One of the four studies that used a complex outcome measurement reported a beneficial effect for the mobility outcome 48. Three of the studies reporting an improvement in either measure only observed this in the subjects who had been weakest and slowest at baseline 47 or in those with pre‐existing low levels of 25(OH)D3 42, 46. In the case of one study 47, no subgroup analysis had been prespecified in the record of the trial registry (clinicaltrials.gov).

### The meta‐analysis

We performed meta‐analyses for the outcomes HGS (kg) and TUG (s).

#### Hand grip strength

The meta‐analysis included seven studies 38, 44, 49, 51, 52, 53, 55, with 767 participants treated with vitamin D and 639 participants treated with control (low‐dose vitamin D or placebo). HGS was measured using various devices and protocols, giving an average HGS at baseline of between 3 and 23 kg. Applying a random effects model, we observed a nonsignificant improvement in HGS after vitamin D supplementation, amounting to 0.2 kg [95% confidence interval (CI) −0.3 to 0.7 kg]. The meta‐analysis revealed significant heterogeneity between the studies (*I* ^2^ = 97%), which was completely eliminated by omitting the three studies that included subjects with vitamin D deficiency 38, 53, 55. After exclusion of these three studies, the effect on the HGS became significant (0.40, 95% CI = 0.37 to 0.43kg). Other sensitivity analyses (Table 1; see also Supporting information, Appendix S4: exclusion of studies using vitamin D_2_, using bolus doses of vitamin D or inclusion of calcium supplements) did not diminish the heterogeneity between the studies and did not change the overall result of a marginal effect of vitamin D supplementation on HGS (Fig. 2).

#### Timed‐up‐and‐go

The meta‐analysis included five studies 45, 47, 49, 50, 52 with 638 participants treated with vitamin D and 622 participants treated with a control or placebo. The studies reported average TUG results ranging from 5 to 11 s. Applying a random effects model, we observed a significant mean increase of 0.3 s in the TUG (95% CI = 0.1 to 0.5 s) after vitamin D supplementation. Thus, the increase would mean a deterioration of the TUG result after vitamin D supplementation. The meta‐analysis revealed significant heterogeneity between the studies (*I* ^2^ = 95%) (Fig. 3). A sensitivity analysis excluding Zhu *et al*. 47 (who used vitamin D_2_ as a supplement and included participants with an average 25(OH)D concentration lower than 50 nmol L^−1^) lead to an insignificant overall estimate of 0.2 s (95% Cl = −0.03 to 0.4s) but did not affect the heterogeneity (sensitivity analysis presented in Table 2; see also Supporting information, Appendix S4).

## Discussion

The objective of this systematic review and meta‐analysis was to investigate whether vitamin D supplementation (with or without calcium) in community‐dwelling older persons can improve muscle strength and mobility. For the present review, 15 RCTs were included for revision, whereas 10 were suitable for the meta‐analysis. Based on findings in nine of the studies, it was concluded that supplementation with vitamin D and/or calcium did not have any beneficial effect on mobility or muscle strength, or on both 34, 38, 43, 44, 48, 49, 51, 52, 53. The main findings of the quantitative meta‐analysis indicated that supplementation with vitamin D did not improve the HGS (based on seven studies) to any significant extent and even had a worsening effect on the TUG‐test results (based on five studies). Therefore, vitamin D supplementation appears to be of limited value for the preservation of muscle strength and mobility in an older population.

### Study population

The older population is heterogeneous in age and the related frailty, as well as with respect to the prevalence of chronic diseases and their treatment, and dependence in the activities of daily life. It can therefore be expected that studies in older persons in general will yield mixed results unless the population is defined more accurately according to the factors mentioned. We therefore limited the present meta‐analysis to community‐dwelling older persons in apparently age‐related good health, although, in many cases, the health status had not been sufficiently well described.

Community‐dwelling older persons are usually in much better health than those hospitalised or living in nursing homes. Targeting these subjects with an intervention aimed at preserving muscle strength and mobility thus appears sensible. However, in concordance with our findings, studies on vitamin D supplementation in hospitalised older subjects 57, 58 or residents in nursing homes 3, 7 showed mixed results for the effects of vitamin D supplementation on muscle strength and mobility. Thus, convincing evidence that vitamin D supplementation may be a useful measure is lacking 14, 28, 59.

Most studies recruited only or predominantly women. Although, at present, there is little evidence that the dietary requirements for vitamin D are different in older men and older women 60, or that the effects of vitamin D on muscle strength are different in older men and women, there is clearly a lack of data on the effect of vitamin D supplementation in men.

### Intervention

Vitamin D exists in two different forms (D_3_ and D_2_). In addition, the inactive form [25(OH)D] and the active form of the hormone [1,25(OH)2D], different routes of administration (oral or intravenous, daily/weekly or bolus supplementation), as well as various doses and various durations of supplementation, can be used. These aspects further complicate comparison of the studies and can introduce heterogeneity between the studies. High‐dose bolus supplementation (either oral or intravenous) has the advantage of high compliance, especially in older subjects who already take a number of medicines on a daily basis. Doses of 300 000 IU are an established treatment for vitamin D deficiency and are regarded as safe. However, doses over 500 000 IU should be avoided because adverse effects of such high doses such as increased falls and fracture risk have been reported 61, 62.

The studies using a low dose were included in the meta‐analysis as a result of studies by Lagari *et al*. 63 and Chao *et al*. 64 stating that 400 IU was inadequate to increase the 25(OH)D concentrations to an acceptable level regardless of baseline 25(OH)D levels in older persons. Because of the high dose of vitamin D_3_ used as a control group in the study by Bischoff‐Ferrari *et al*. 42, we choose not include the study in our meta‐analysis.

### Outcomes

The functional improvement in the older persons has been measured using a range of measurements employing different protocols. Among these, the HGS has been shown to be a reliable parameter 65, 66 for long‐term health outcomes. There is, however, less evidence for the TUG test for long‐term health outcomes.

We included only quantifiable outcomes in the present study but not falls or fractures that have been used as measure of reduced muscle strength and as clinical outcomes in other studies 15, 67. However, determining falls may be difficult in community‐dwelling older persons because it relies heavily on the subjects' recall and may thus reduce the reliability of this outcome.

We observed a small and nonsignificant improvement in HGS as a result of vitamin D supplementation in the meta‐analysis, which was also characterised by a high degree of heterogeneity between studies. However, the magnitude of the effect may also indicate that other health measures, such as exercise and potentially supplementation with other nutrients, should be prioritised. In addition, the huge variation in baseline HGS measurements between studies further complicates the interpretation of the effects of high/increased vitamin D intake. The nonsignificant result may be regarded as contradicting observational studies in community‐dwelling older persons because other studies have reported that a doubling of the 25(OH)D concentration from 50 to 100 nmol L^−1^ was associated with a higher HGS in men and in women, with increases of approximately 4.4 and 0.8 kg, respectively 68. We observed a significant and stronger improvement of HGS and diminished heterogeneity after the exclusion of three studies with low 25(OH)D concentrations at baseline 38, 53, 55. Low vitamin D status may reflect a higher degree of frailty 69, and supplementation may therefore be too late to improve muscle strength in those with very low 25(OH)D levels, despite the correction of vitamin D deficiency as indicated by the serum levels.

Although the effect of vitamin D supplementation on the TUG test suggests a negative direction by increasing the time used for the test, this result should be taken with caution because the meta‐analysis showed a high degree of heterogeneity that was not removed by excluding single studies (Table 2; see also Supporting information, Appendix S4). In addition, the overall magnitude of the effect was very small, suggesting that this change is clinically less meaningful. Overall, the small number of studies and the high degree of heterogeneity precludes any firm conclusions, although further investigations are certainly warranted. It would be interesting to determine whether interventions combined with exercise and/or other nutrients would improve the test outcome. This has already been shown by Bunout *et al*. 12, who used exercise and vitamin D supplements as interventions in vitamin D‐deficient community‐dwelling older persons and observed a positive effect on TUG.

The importance of calcium supplements should also be considered. Because of the concurrent administration of calcium in most and especially in the larger studies 43, 45, 46, 47, 49, 52, 53, 55, it is impossible to determine any independent effect of either vitamin D or calcium. The use of calcium supplements for purposes other than improvement of bone health has been strongly debated. It is also plausible to combine vitamin D supplements with calcium because vitamin D increases calcium absorption from the gut but, in the case of insufficient dietary calcium intake, this can also affect bone remodelling 70.

### Comparison with previous systematic reviews and meta‐analyses

The effect of vitamin D on physical performance has been summarised in systematic reviews 28, 30, 33 and in three meta‐analyses 29, 31, 32. These investigations are characterised by either including all age groups 30, 31, 32, by including older adults from different settings (community‐dwelling and institutionalised, 14, 28, 29, different study designs 33 and investigating composite outcomes 32, thus making comparisons with our findings difficult. Stockton *et al*. 31 also reported a meta‐analysis for HGS and, in line with our findings, reported no significant effect on HGS. The only other meta‐analysis that reported TUG as an outcome reported a small, significant improvement of this test, based on three studies 29.

Thus, the overall results are difficult to compare, although they demonstrate the large number of tests used for the assessment of muscle strength, physical performance and mobility. A common test battery would make comparisons between studies much easier.

### Strengths and limitations

The strengths of this review include the use of data from 15 RCTs, with approximately 2800 participants treated with vitamin D or a control, and the analysis of quantitative outcomes such as HGS and TUG, which have been shown to be related to other clinical outcomes in the older persons 71.

The main limitation of this review is the small number of studies available for the meta‐analysis, mainly as a result of heterogeneity of the measurements used. Another limitation is the variation in study populations, with a wide range of comorbidities. More exact descriptions of the population under study are urgently needed to improve comparability of studies and to increase external validity. We also observed heterogeneity between studies that could not be resolved by subgroup analyses.

In conclusion, we observed no improvement in muscle strength after administration of vitamin D with or without calcium supplements. We did find a small but significant deterioration of mobility. This is, however, based on a limited number of studies and participants.

## Transparency declaration

The lead author affirms that this manuscript is an honest, accurate and transparent account of the study being reported, that no important aspects of the study have been omitted and that any discrepancies from the study as planned (and registered with) have been explained. The reporting of this work is compliant with CONSORT^1^/STROBE^2^/PRISMA^3^ guidelines.

**Conflict of interest, source of funding and authorship**

The authors declare that they have no conflicts of interest.

Funding support was provided by the Norwegian Seafood and Research Fund (FHF). This is the correct funding. The sponsor had no role in the paper.

HRR and JD designed the study. HRR, US and JD performed the literature search and the meta‐analysis. All authors read the included papers and were substantially involved in the writing process. All authors critically reviewed the manuscript and approved the final version submitted for publication.

## Supporting information


**Appendix S1.** Search terms and search hits in Medline, Embase, Pubmed and Web of Science.
**Appendix S2.** An overview of the physical performance tests used in the randomised controlled trials included in the systematic review and meta‐analysis.
**Appendix S3.** Summary of CONSORT statements for each study included in the systematic review.
**Appendix S4.** Sensitivity analysis for hand grip strength (HGS) and timed‐up‐and‐go test (TUG).
