# Supplementary material for: Vitamin D supplementation and its influence on muscle strength and mobility in community‐dwelling older persons: a systematic review and meta‐analysis
Source: J Hum Nutr Diet. 2016 Jul 27;30(1):3–15. doi: 10.1111/jhn.12394 (PMC5248635; doi:10.1111/jhn.12394)
Supplement: Supplementary file 1 — Appendix S1. Search terms and search hits in Medline, Embase, Pubmed and Web of Science. Appendix S2. An overview of the physical performance tests used in the randomised controlled trials included in the systematic review and meta‐analysis. Appendix S3. Summary of CONSORT statements for each study included in the systematic review. Appendix S4. Sensitivity analysis for hand grip strength (HGS) and timed‐up‐and‐go test (TUG). [file JHN-30-3-s001.docx]

SUPPLEMENTAL MATERIAL FOR ONLINE ONLY

Appendix S1: Search terms and search hits in Medline, Embase, Pubmed and Web of Science.

| **Steps** | **Search term** | **Search string** | **Hits Medline** | **Hits Embase** | **Hits**  **PubMed** | **Hits Cochrane Library** | **Hits Web of Science** |
| --- | --- | --- | --- | --- | --- | --- | --- |
| 1 | Vitamin D | “vitamin d [MeSH Terms]” OR “vitamin d [All fields]” |  |  |  |  |  |
| 2 | 25-OH-D | “25-OH-D [All fields]” |  |  |  |  |  |
| 3 | Calcidiol | “calcidiol {All fields]” |  |  |  |  |  |
| 4 | Cholecalciferol | “cholecalciferol {All fields]” |  |  |  |  |  |
| 5 | Calciferol | “calciferol [All fields]” |  |  |  |  |  |
| 6 | ergocalciferol | «ergocalciferol [All fields]» |  |  |  |  |  |
| 7 | hydroxycholecalciferols | “hydroxycholecalciferols {All fields]” |  |  |  |  |  |
| 8 | 1,25-dihydroxyvitamin d2 | “1,25-dihydroxyvitamin d2 [All fields]” |  |  |  |  |  |
| 9 | dihydroxycholecalciferol | “dihydroxycholecalciferols [All fields]” |  |  |  |  |  |
| 10 | 1 or 2 or 3 or 4 or 5 or 6 or 7 or 8 or 9 |  |  |  |  |  |  |
|  |  |  |  |  |  |  |  |
| 11 | Muscle strength | “Muscle strength [MeSH Terms]” |  |  |  |  |  |
| 12 | Hand strength | “Hand strength [All fields]” |  |  |  |  |  |
| 13 | Weakness | “Weakness [All fields]” |  |  |  |  |  |
| 14 | Strength | “Strength [All fields]” |  |  |  |  |  |
| 15 | Gait | “gait {MeSH Terms]” |  |  |  |  |  |
| 16 | Mobility | “Mobility [All fields]” |  |  |  |  |  |
| 17 | 11 or 12 or 13 or 14 or 15 or 16 |  |  |  |  |  |  |
| 188 | Aging | «Aging {MeSH Terms]”» |  |  |  |  |  |
| 19 | Aged | «Aged {MeSH Terms]”» |  |  |  |  |  |
| 20 | Frail elderly | «frail elderly {All fields]”» |  |  |  |  |  |
| 21 | Elderly | “Elderly [All fields]” |  |  |  |  |  |
| 22 | Older adults | “Older adults [All fields] |  |  |  |  |  |
| 23 | 17 or 18 or 19 or 20 or 21 or 22 |  |  |  |  |  |  |
| 24 | 10 and 17 and 23 |  |  |  |  |  |  |
| **8** | **Results** |  | **747(109RCT)** | **1554(166RCT)** | **959(115RCT)** | **224 (193 trials)** | **1040** |

Appendix S2: An overview of the physical performance tests used in the RCTs included in the systematic review and meta-analysis.

| **Physical performance test** | **Short description** |
| --- | --- |
| Hand grip strength (HGS) | Hand grip strength was measured with a hand-held dynamometer ^43, 45, 46^. The average of two measurements was used for the analysis (recorded to the nearest half kilogram of force) ^45^. |
| Timed up and go (TUG) | Subjects were observed and timed from the instant they rose from an armchair (seat height 48 cm, arm height 68 cm), walked 3 or 4 meters, navigated an obstacle on the floor, and returned to a fully seated position in the chair. Each subject performed the test three times and the fastest of the three trials was finally recorded [41, **1**]. |
| Quadriceps strength | Quadriceps strength was assessed using a strain gauge system attached to a specially constructed chair on which the participants were seated with their hips and knees flexed to approximately 90°. In this position, three maximal voluntary contractions (MVC) were recorded and analysed off-line. The strongest MVC of the dominant leg was used in the data analysis [43, **2**]. |
| Knee extension | Subjects were instructed to complete 5 knee extension repetitions, each separated by 30 seconds, as quickly as possible through their full range of motion at both 40% and 70% of one repetition maximum [28]. |
| Leg muscle strength | Isokinetic muscle strength of the knee flexor and extensor muscles was measured using an isokinetic dynamometer [40]. |
| Gait speed | Subjects completed a 4-meter walk test along a corridor at their normal walking speed. Gait speed was calculated by dividing the 4 meters by the time in seconds taken to complete the test [37]. |
| Stair climbing power | The subject was asked to climb a flight of stairs (10 steps, 7.8 cm rise/step) as quickly as possible. It should be done without the use of handrails or any other aid [46]. The stair climbing power was calculated was calculated according to the method by Lazowski et al. [**3**] |
| The four square step test (FSST) | A square is formed by using 4 canes resting flat on the floor. The subject stands in square number 1 facing square number 2. The aim is to step as fast as possible into each square in the following sequence; square number 2, 3, 4, 1, 4, 3, 2, and 1.The time spent on finishing the sequence is recorded and a score is given according to time spent [**4**]. |
| Leg extension power (LEP) | LEP was measured using a Notting Power Rig. The subject, in seated position with folded arm and a 90-degree knee angle at the start pushed a large foot pedal as hard and fast as possible, setting a flywheel in motion. The best of seven measurements was recorded on both sides [45, **5**]. |
| Modified coop test (COOP) | The COOP is a test in which the maximum walking distance (in meters) achieved in 2 minutes is recorded [45, **6**]. |
| Lower limb muscle strength | Ankle dorsiflexion, knee flexor, knee extensor, hip abductor, hip flexor, hip extensor, and hip adductor strengths were assessed using a strain gauge. The subjects were requested to exert maximum muscle contraction against the strain gauge. The best of three attempts was recorded for each muscle group [44]. |
| Five-times-sit-to-stand-test (FTFFT) or Repeated sit-to-stand-test | Using a chair of standard height (40-45 cm), the subjects stood up and sat down five times as rapidly as possible, with the arms folded across the chest. Timing with a digital stopwatch began with the command “go” and ceased when the subject stood up the last time. Two trials were allowed, and the subjects were allowed sufficient time between trials for complete recovery [47, **7**]. The repeated sit-to-stand-test is in principal the same [39]. |
| Short Physical Performance Battery (SPPB) | SPPB includes an assessment of standing balance, a gait speed test (i.e. a timed 4-m walk), and timed rising from a chair and sitting without the use of arms, with 5 repetitions. The SPPB was evaluated using a point scale running from 0 to 12 for combined measures of balance, gait speed, and the ability to rise from a sitting position in a chair, or separately as a continuous variable for the gait speed test [36, **8**]. |
| Functional performance (APFT) | Assessment of lower limb functional performance by determining the time taken to perform four common activities of daily living (50 ft walk, rising from a 42 cm high chair and walking 50 ft, going up and down 13 steps). The sum of the times (in seconds) taken to perform these activities was calculated to determine the Aggregate Functional Performance Time (AFPT) [43, **9**]. |
| Knee flexion strength | Knee flexor strength was measured in kiloponds, with a validated, hand-held  isometric method (model DPPH; Chatillon Inc., Greensboro, NC, USA). A nonelastic band was connected to a pull gauge with a continues scale in kiloponds (1 kilopond=10  Newton) [**10**]. |

References only used in the appendix (in bold writing):

1. Podsiadlo, D. and S. Richardson, *The timed "Up & Go": a test of basic functional mobility for frail elderly persons.* J Am Geriatr Soc, 1991. **39**(2): p. 142-8.
2. Edwards, R.H., et al., *Human skeletal muscle function: description of tests and normal values.* Clin Sci Mol Med, 1977. **52**(3): p. 283-90.
3. Lazowski DA, Ecclestone NA, Myers AM, Paterson DH, Tudor-Locke C, Fitzgerald C, et al. A randomized outcome evaluation of group exercise programs in long-term care institutions. The journals of gerontology Series A, Biological sciences and medical sciences. 1999;54(12):M621-8.
4. Dite W, Temple VA. A clinical test of stepping and change of direction to identify multiple falling older adults. Archives of physical medicine and rehabilitation. 2002;83(11):1566-71.
5. Bassey, E.J. and A.H. Short, *A new method for measuring power output in a single leg extension: feasibility, reliability and validity.* Eur J Appl Physiol Occup Physiol, 1990. **60**(5): p. 385-90.
6. Butland, R.J., et al., *Two-, six-, and 12-minute walking tests in respiratory disease.* Br Med J (Clin Res Ed), 1982. **284**(6329): p. 1607-8.
7. Whitney, S.L., et al., *Clinical measurement of sit-to-stand performance in people with balance disorders: validity of data for the Five-Times-Sit-to-Stand Test.* Phys Ther, 2005. **85**(10): p. 1034-45.
8. Guralnik, J.M., et al., *A short physical performance battery assessing lower extremity function: association with self-reported disability and prediction of mortality and nursing home admission.* J Gerontol, 1994. **49**(2): p. M85-94.
9. Hurley, M.V., J. Rees, and D.J. Newham, *Quadriceps function, proprioceptive acuity and functional performance in healthy young, middle-aged and elderly subjects.* Age Ageing, 1998. **27**(1): p. 55-62.
10. Bischoff HA, Stähelin HB, Dick W, Akos R, Knecht M, Salis C, et al. Effects of vitamin D and calcium supplementation on falls: a randomized controlled trial. Journal of bone and mineral research : the official journal of the American Society for Bone and Mineral Research [Internet]. 2003; 18(2):[343-51 pp.]

Appendix S3: Summary of CONSORT statements for each study included in the systematic review

| **Section/topic** | **Item no** | **Checklist item** | Bischoff-Ferrari, 2012 | Ceglia, 2013 | Dhesi, 2004 | Glendenning, 2012 | Grady, 1991 | Janssen, 2009 | Kenny, 2003 | Lagari, 2010 | Lips, 2010 | Pfeifer, 2009 | Pirotta, 2015 | Songpatansilp, 2009 | Wood, 2014 | Xia, 2009 | Zhu, 2010 |
| --- | --- | --- | --- | --- | --- | --- | --- | --- | --- | --- | --- | --- | --- | --- | --- | --- | --- |
| **Title and abstract** |  |  |  |  |  |  |  |  |  |  |  |  |  |  |  |  |  |
|  | 1a | Identification as a randomised trial in the title | - | + | - | + | + | + | - | - | - | - | + | + | + | - | + |
|  | 1b | Structured summary of trial design, methods, results and conclusions | + | +/- | + | + | + | + | + | + | + | + | + | + | + | + | + |
| **Introduction** |  |  |  |  |  |  |  |  |  |  |  |  |  |  |  |  |  |
| Background and objectives | 2a | Scientific background and explanation of rationale | + | + | + | + | + | + | + | + | + | + | + | + | + | + | + |
|  | 2b | Specific objectives or hypotheses | + | + | + | + | + | + | + | + | - | + | + | + | + | + | + |
| **Methods** |  |  |  |  |  |  |  |  |  |  |  |  |  |  |  |  |  |
| Trial design | 3a | Description of trial design including allocation ratio | + | + | + | + | - | - | + | + | + | - | + | + | + | + | + |
|  | 3b | Important changes to methods after trial commencement (such as eligibility criteria), with reasons | - | - | - | - | - | - | - | - | - | - | - | - | - | - | - |
| Participants | 4a | Eligibility criteria for participants | + | + | + | + | + | + | + | + | + | + | + | + | + | + | + |
|  | 4b | Settings and locations where the data were collected | + | + | + | + | - | + | + | + | + | + | + | + | + | + | + |
| Interventions | 5 | The interventions for each group with sufficient details to allow replication, including how and when they were actually administered | + | + | + | + | - | + | + | + | + | + | + | + | + | + | + |
| Outcomes | 6a | Completely defined pre-specified primary and secondary outcome measures, including how and when they were assessed | + | + | + | + | + | + | + | + | + | + | + | + | + | + | + |
|  | 6b | Any changes to trial outcomes after the trial commenced, with reasons | - | - | - | - | - | - | - | - | - | - | - | - | - | - | - |
| Sample size | 7a | How sample size was determined | - | + | - | + | - | - | - | - | + | - | + | - | + | + | + |
|  | 7b | When applicable, explanation of any interim analyses and stopping guidelines | - | + | - | - | + | - | - | - | + | - | - | - | - | - | - |
| Randomization: |  |  |  |  |  |  |  |  |  |  |  |  |  |  |  |  |  |
| Sequence | 8a | Method used to generate the random allocation sequence | - | - | + | + | - | + | - | + | - | - | + | - | + | + | + |
| Generation | 8b | Type of randomisation; details of any restriction (such as blocking and block size) | - | - | + | + | - | + | - | + | - | - | - | - | - | + | + |
| Allocation concealment mechanism | 9 | Mechanism used to implement the random allocation sequence (such as sequentially numbered containers), describing any steps taken to conceal the sequence until interventions were assigned | - | - | - | + | - | + | + | + | - | - | + | + | + | + | + |
| Implementation | 10 | Who generated the random allocation sequence, who enrolled participants, and who assigned participants to interventions | - | - | - | + | - | + | + | - | - | - | + | + | + | + | + |
| Blinding | 11a | If done, who was blinded after assignment to interventions (for example, participants, care providers, those assessing outcomes) and how | + | - | - | + | - | + | + | - | - | - | + | + | + | - | + |
|  | 11b | If relevant, description of the similarity of interventions | + | NR | NR | NR | NR | NR | NR | NR | NR | NR | NR | NR | NR | NR | NR |
| Statistical methods | 12a | Statistical methods used to compare groups for primary and secondary outcomes | + | + | + | + | + | + | + | + | + | + | + | + | + | + | + |
|  | 12b | Methods for additional analyses, such as subgroup analyses and adjusted analyses | + | + | - | + | - | + | - | + | + | + | + | + | + | - | + |
| **Results** |  |  |  |  |  |  |  |  |  |  |  |  |  |  |  |  |  |
| Participant flow | 13a | For each group, the numbers of participants who were randomly assigned, received intended treatment, and were analysed for the primary outcome | + | + | + | + | + | + | + | + | + | + | + | - | + | + | + |
|  | 13b | For each group, losses and exclusions after randomisation, together with reasons | - | + | + | + | + | + | + | + | + | + | + | + | + | + | + |
| Recruitment | 14a | Dates defining the periods of recruitment and follow-up | - | - | + | + | - | - | - | - | + | - | - | - | + | - | - |
|  | 14b | Why the trial ended or was stopped | NR | NR | NR | NR | NR | NR | NR | NR | NR | NR | NR | NR | NR | NR | NR |
| Baseline data | 15 | A table showing baseline demographic and clinical characteristics for each group | + | + | + | + | + | + | + | + | + | + | + | + | + | + | + |
| Numbers analysed | 16 | For each group, number of participants (denominator) included in each analysis and whether the analysis was by original assigned groups | - | + | - | + | + | + | + | + | + | + | - | - | + | + | + |
| Outcomes and estimation | 17a | For each primary and secondary outcome, results for each group, and the estimated effect size and its precision (such as 95% confidence interval) | + | + | + | + | + | + | + | + | + | + | + | + | + | + | + |
|  | 17b | For binary outcomes, presentation of both absolute and relative effect sizes is recommended | - | - | - | - | - | - | - | - | - | - | - | - | - | - | - |
| Ancillary analyses | 18 | Results of any other analyses performed, including subgroup analyses and adjusted analyses, distinguishing pre-specified from exploratory | - | - | - | + | + | - | - | + | + | + | - | + | - | + | + |
| Harms | 19 | All important harms or unintended effects in each group (for specific guidance see CONSORT for harms) | - | + | - | + | + | + | - | - | + | - | - | + | + | + | + |
| **Discussion** |  |  |  |  |  |  |  |  |  |  |  |  |  |  |  |  |  |
| Limitations | 20 | Trial limitations, addressing sources of potential bias, imprecision, and, if relevant, multiplicity of analyses | + | + | - | + | + | + | + | + | + | + | + | + | + | + | + |
| Generalizability | 21 | Generalisability (external validity, applicability) of the trial findings | + | + | + | + | + | + | + | + | + | + | + | + | + | + | + |
| Interpretation | 22 | Interpretation consistent with results, balancing benefits and harms, and considering other relevant evidence | - | + | + | + | + | + | + | + | + | + | + | + | + | + | + |
| **Other information** |  |  |  |  |  |  |  |  |  |  |  |  |  |  |  |  |  |
| Registration | 23 | Registration number and name of trial registry | + | - | - | + | - | - | - | - | + | - | + | - | + | - | + |
| Protocol | 24 | Where the full trial protocol can be accessed, if available | - | + | - | - | - | - | - | + | + | - | - | - | + | - | + |
| Funding | 25 | Sources of funding and other support (such as supply of drugs), role of funders | + | + | + | + | + | + | + | + | + | + | + | + | + | + | + |

+ = stated, - = not stated, NR= not relevant

Appendix S4: Sensitivity analysis for hand grip strength (HGS) and timed up and go test (TUG)

Table 1: Sensitivity analysis for handgrip strength (HGS, kg). The studies mention in the parenthesis is excluded from the analysis.

Table 2: Sensitivity analysis for timed up and go test (TUG, s). The studies mention in the parenthesis is excluded from the analysis.
